# Increased risk of acute stress disorder and post-traumatic stress disorder in children and adolescents with autism spectrum disorder: a nation-wide cohort study in Taiwan

**DOI:** 10.3389/fpsyt.2024.1329836

**Published:** 2024-01-31

**Authors:** Sung-Tao Li, Wu-Chien Chien, Chi-Hsiang Chung, Nian-Sheng Tzeng

**Affiliations:** ^1^Department of Psychiatry, Tri-Service General Hospital, School of Medicine, National Defense Medical Center, Taipei, Taiwan; ^2^Department of Psychiatry, Hualien Armed Forces General Hospital, Hualien, Taiwan; ^3^Department of Medical Research, Tri-Service General Hospital, National Defense Medical Center, Taipei, Taiwan; ^4^School of Public Health, National Defense Medical Center, Taipei, Taiwan; ^5^Taiwanese Injury Prevention and Safety Promotion Association, Taipei, Taiwan; ^6^Graduate Institute of Life Sciences, National Defense Medical Center, Taipei, Taiwan; ^7^Student Counseling Center, National Defense Medical Center, Taipei, Taiwan

**Keywords:** child and adolescent psychiatry, autism spectrum disorder, acute stress disorder, post-traumatic stress disorder, epidemiology

## Abstract

**Introduction:**

Children and adolescents with autism spectrum disorder (ASD) may be particularly vulnerable to the impact of traumatic events, yet the association between ASD and the risk of developing acute stress disorder and post-traumatic stress disorder (PTSD) remains uncertain. This study aims to investigate this association, addressing the gap in large-scale evidence on the subject.

**Methods:**

Conducted as a retrospective and matched cohort study, data was sourced from the National Health Insurance Research Database (NHIRD) in Taiwan, spanning from January 1, 2000, to December 31, 2015. The study included patients aged 18 years or under newly diagnosed with ASD (n=15,200) and compared them with a matched control group (n=45,600). The Cox proportional regression model was employed to assess the risk of acute stress disorder and PTSD.

**Results:**

Over the 15-year follow-up period, a total of 132 participants developed either acute stress disorder or PTSD. Among them, 105 cases (0.691% or 64.90 per 100,000 person-years) were in the ASD group, while 27 cases (0.059% or 5.38 per 100,000 person-years) were in the control group. The adjusted hazard ratio for the ASD group was significantly higher compared to the control group (25.661 with 95% CI = 15.913-41.232; P < .001).

**Discussion:**

This study provides compelling evidence that individuals with ASD face an elevated risk of developing acute stress disorder and PTSD. The findings underscore the importance of clinicians recognizing and addressing this vulnerability in ASD individuals exposed to traumatic events. This emphasizes the need for heightened attention to the risk of PTSD and acute stress disorder in the ASD population.

## 1 Introduction

Autism spectrum disorder (ASD) is a neurodevelopmental disorder, characterized by persistent social communication and interaction deficits, combined with restricted and repetitive behavior. Recent studies have indicated that the worldwide prevalence of ASD was approximately 1% to 2%. Moreover, ASD is considered to be approximately two to three times more prevalent among males than females, with differences among countries (1). In Taiwan, the pooled prevalence of ASD was around 0.26% (2). Higher rates of psychiatric comorbidities in ASD individuals have been reported (3), with attention deficit/hyperactivity disorder (ADHD), anxiety, and depression being the most common comorbidities (4). Children and adolescents with ASD may be vulnerable to psychosocial frustration, traumatic events (5), and increased risks of mood symptoms and suicide ideation (6) or suicide attempt (7, 8).

Post-traumatic stress disorder (PTSD) is characterized by the symptomology of intrusive re-experience, avoidance, hyperarousal, and marked negative change in mood and cognition after exposure to traumatic events, defined as exposure to actual death or threatened death, severe injury, or sexual violence (9, 10). Previous studies conducted in Taiwan, focused mainly on adult groups have revealed that PTSD is mutually associated with diseases such as obstructive sleep apnea (11); asthma (12); hypertension, diabetes, and dyslipidemia (13); osteoporosis (14); epilepsy (7, 8); Parkinson’s disease (15); dementia(16); and substance use disorder (17). The risk of experiencing traumatic events is higher in ASD individuals than in typically developed peers (18). Mood symptomatology, including anxiety and depression, had the strongest link to trauma exposure in ASD youth (19) and also PTSD is often co-exiting with symptoms of depression and anxiety (20). In addition, growing evidence has revealed that the symptomatology of PTSD was observed in ASD individuals with traumatic exposure (21, 22). In children and adolescents with ASD, PTSD seems to develop at a comparable or higher rate when compared to the general population, with prevalence estimated from 0% to approximately 17% in a systemic review (23). On the contrast, in a study with the general population of 1,420 children with trauma exposure, less than 0.5% of the children fulfill the criteria of full-blown PTSD diagnosis (24). To date, the prevalence estimate PTSD in the ASD population remains mostly unknown, without large-scaled population-based studies assessing prevalence (23).

Acute stress disorder is characterized by a cluster of symptoms as the diagnosis of PTSD in the acute phase within one month after trauma exposure (9, 10). Nonetheless, previous studies have claimed that a diagnosis of acute stress disorder may be limited in appropriately predicting individuals who will later develop PTSD (25, 26), including children and adolescents (27, 28). However, the presentation and prevalence of acute stress disorder in the ASD group is yet to be clarified.

Previous studies have revealed that individuals with ASD are at a higher risk of exposure to traumatic life events and of developing PTSD (5, 18, 29). Most previous studies are case reports, case series, cross-sectional observational researches, or systemic reviews (23). A previous cohort study used a questionnaire to define the autistic traits of women with children. The adjusted odds ratio (aOR) of PTSD was significantly higher in the top three quintiles of questionnaires (aOR = 1.4 to 1.9). Furthermore, the association between autistic traits and PTSD was identified (30). Some previous studies have administered questionnaires so as to define autistic traits or various types of structural diagnostic tools of ASD.

Several previous studies have investigated the association of ASD between type 2 diabetes mellitus (31), suicide attempts (7, 8), and substance use disorders (17), and PTSD (23). In addition, some peri-natal or early childhood clinical conditions, such as neonatal hyperbilirubinemia (32), being born prematurely (33), and exposure of general anesthesia (34), are associated with a higher risk of ASD. However, the association among ASD, acute stress disorder, and PTSD is yet to be clarified. We hypothesized that children and adolescents with ASD are at a higher risk of developing acute stress disorder and PTSD. Therefore, we conducted this nationwide population-based cohort study so as to determine the risk of acute stress disorder and PTSD in individuals with ASD in Taiwan.

## 2 Methods

### 2.1 Data sources

We used the data from the National Health Insurance Research Database (NHIRD) in Taiwan to investigate the association of ASD with PTSD and acute stress disorder over a 15-year period. The National Health Insurance (NHI) program was begun in 1995, and as of June 2009, the program included contracts with 97% of medical providers in Taiwan. Approximately, 23 million beneficiaries, that is, more than 99% of the entire population have been registered in the NHIRD (35). The information in the NHIRD is stored separately in different sub-datasets, including registry for beneficiaries, registry for medical facilities, registry for board-certified specialists, inpatient claims, ambulatory care claims, and prescriptions dispensed at pharmacies. Demographic features in the patient-level information are provided by the linkage between these datasheets and individual personal identification numbers. The diagnosis recorded in the NHIRD was based on the *International Classification of Diseases, 9^th^ Revision, Clinical Modification* (ICD-9-CM) codes. The psychiatric diagnosis was established by certified psychiatrists, according to the *Diagnostic Statistical Manual of Mental Disorders, 4^th^ Edition* and the revised edition (9, 10) (**Supplementary Table S1**). The validation of the NHRID has been discussed, and several studies have been investigating the validity of the diagnosis codes, with modest to high sensitivity and positive predictive values. (36–39.). In Taiwan, patients with the catastrophic illness certification, who get care for the illness or it’s related conditions, do not need to pay a co-payment for outpatient or inpatient care, and ASD is one of the catastrophic illnesses (40). Therefore, the patients with ASD, or their parents, could apply for catastrophic illness certification under the NHI program, including our aim of autism spectrum disorder, of the ICD-9-CM code: 299. Since the diagnosis with catastrophic illness certification would have been reviewed by experts and the status of catastrophic illness certification would be with high accuracy for ASD in the NHIRD.

### 2.2 Study design and sample

The diagnosis of ASD was used after the publication of the *Diagnostic Statistical Manual of Mental Disorders, 5^th^ Edition* (DSM-5) in 2013 (41), which included previous diagnoses in the DSM-IV-TR, as Asperger’s disorder, and pervasive developmental disorder, not otherwise specified. This study employed a retrospective, population-based, and matched cohort design, adhering to ICD-9-CM code: 299, autistic disorder in the NHIRD data to represent the ASD.

**Figure 1:**
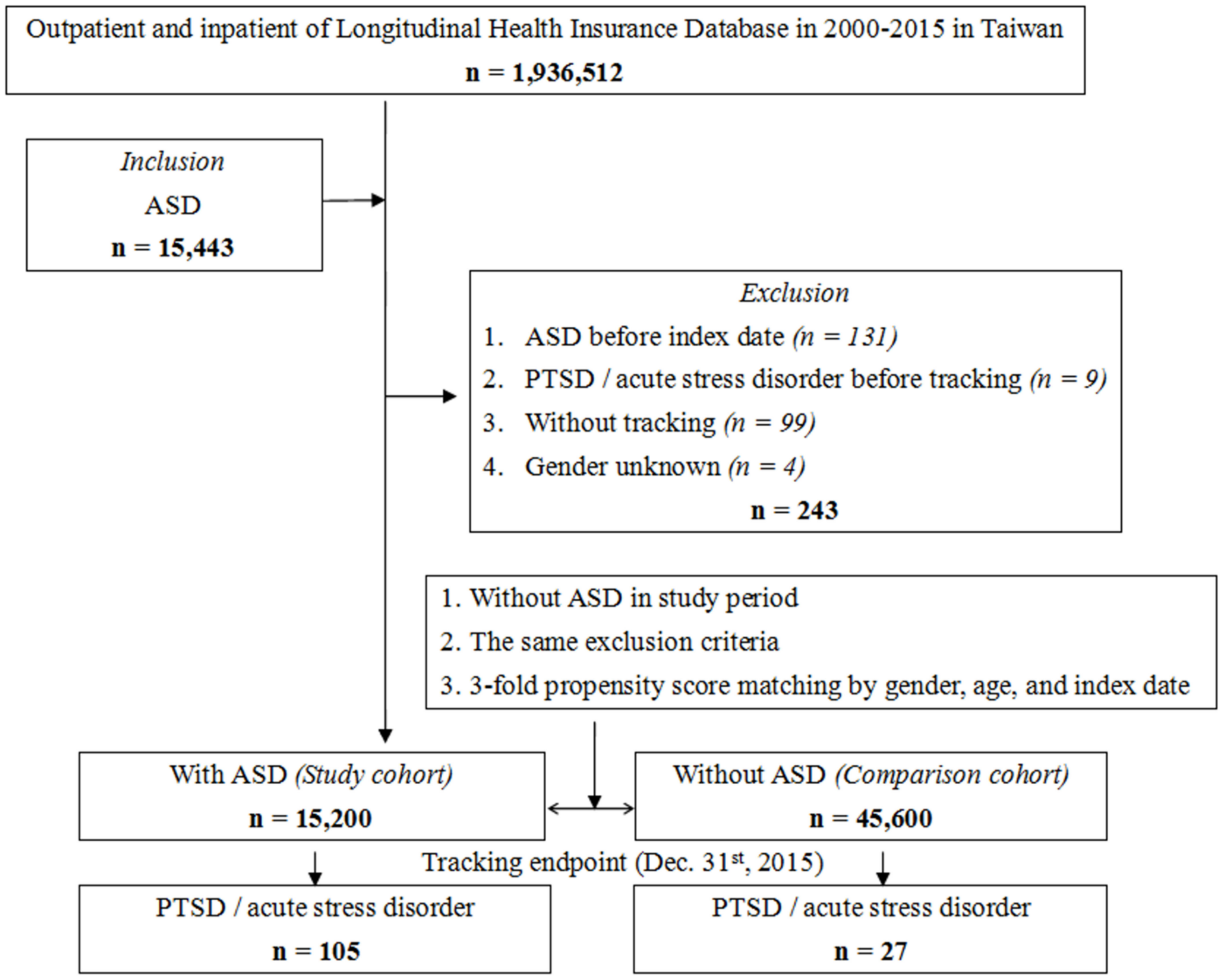
Flowchart of study sample selection from National Health Insurance Research Database in Taiwan.

The subject selection process is presented in **Figure 1**. The cohort comprised children and adolescents aged 18 years or under who received a new ASD diagnosis (ICD-9-CM codes: 299) between January 1, 2000, and December 31, 2015. A total of 15,200 individuals with ASD were enrolled, and a control group of 45,600 individuals without ASD was selected, matched for sex, age, and index date at a 1:3 ratio. Recognizing the higher prevalence of ASD in males and the diverse age at diagnosis, we chose to match age and gender to address potential confounding factors. The rationale for conducting case/control matching in a 1:3 ratio can be observed in **Supplementary Figure S1**. **Supplementary Figure S1A** illustrates that a control/case ratio of 1:3 achieves a test power exceeding 97.5%, acknowledging the impracticality of an unlimited increase in controls. In **Supplementary Figure S1B**, using a 1:3 ratio results in an estimated power approaching 1.0, prompting our selection of this ratio for optimal statistical power in our study. The index date was defined as the ASD diagnosis event certified by psychiatrists. Participants diagnosed with ASD, acute stress disorder, or PTSD before 2000 were excluded. Follow-up extended from the index date until the diagnosis of PTSD (ICD-9-CM code: 309.81) or acute stress disorder (ICD-9-CM code: 308) by certified psychiatrists.

Urbanization levels were determined based on population size and developmental indicators. Level 1 urbanization included areas with a population exceeding 1,250,000 and specific designations for cultural, economic, political, and metropolitan development. Level 2 comprised areas with a population between 500,000 and 1,249,999, playing significant roles in culture, economy, and politics. Urbanization levels 3 and 4 included populations ranging from 149,999 to 499,999 and less than 149,999, respectively.

Comorbidities were assessed using the Charlson Comorbidity Index (CCI), calculated from ICD-9-CM codes of each individuals in the original data of NHIRD with scores for each comorbidity category. A score of zero denoted the absence of comorbidities, with higher scores indicating a greater comorbidity burden. Additional psychiatric comorbidities considered included intellectual disability (ICD-9-CM code: 317-319, V62.89), childhood emotional disorders (ICD-9-CM code: 313), ADHD (ICD-9-CM code: 314), Tourette syndrome/tics disorders (ICD-9-CM code: 307.2), conduct disorder/oppositional defiant disorder (ICD-9-CM code: 312), other developmental disorders (ICD-9-CM code: 315), enuresis/encopresis (ICD-9-CM code: 307.6-307.7), and injury (ICD-9-CM code: 800-999, E800-E999) (42). The ICD-9-CM codes for diagnoses and clinical situations in this study are presented in **Supplementary Table S1**.

### 2.3 Statistical analysis

Complete analysis was performed using the software SPSS version 22.0 (IBM SPSS Statistics for Windows, Version 22.0, IBM Corp, Armonk, NY, USA) χ2⁠ and *t* tests were applied to evaluate the distributions of the categorical and continuous variables, respectively. The Fisher exact test was used to evaluate the differences in categorical variables between the study and control group. The Cox proportional-hazard regression analysis was applied to determine the risk of PTSD and acute stress disorder, and results are presented as a hazard ratio with a 95% confidence interval. The difference in the risk between the ASD and the control groups were determined using the Kaplan–Meier method with the log-rank test. A 2-tailed *P* value of <.05 was considered to indicate statistical significance. A sensitivity analysis was performed to assess the persistence of associations following the exclusion of diagnoses for acute stress disorder and/or PTSD within the initial year and the first five years.

## 3 Results

### 3.1 Baseline characteristics

**Figure 2:**
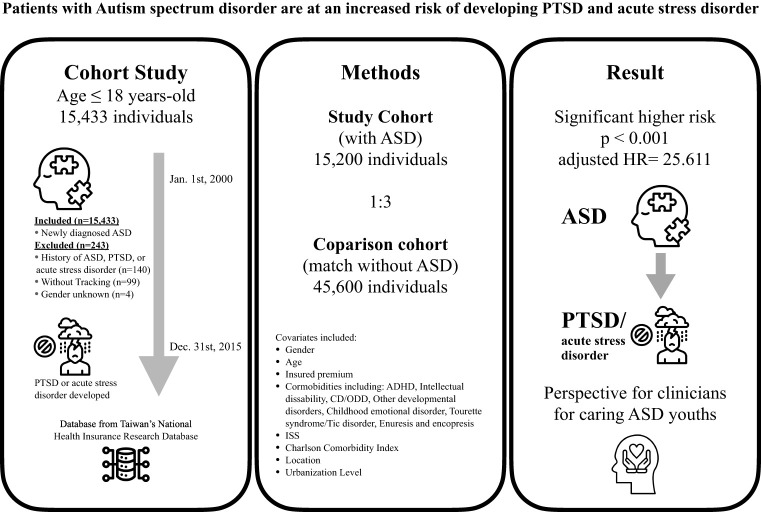
The graphic abstract of study design and results from National Health Insurance Research Database in Taiwan. ASD, Autism spectrum disorder; PTSD, Post-traumatic stress disorder; ADHD, Attention-deficit hyperactivity disorder; CD, Conduct disorder; ODD, Oppositional defiant disorder; ISS, Injury Severity Scale; HR, Hazard ratio. All icons are from the Noun Project.

**Table 1** presents the study population, and **Figure 2** of the graphic abstract provides an illustration for the study design and results. Analysis revealed significant differences in age and gender between the ASD case group and the original unmatched group. (**Supplementary Table S3**) Matching on gender and age facilitated reduced confounding, improved comparability, and increase the power to 0.999 compared to unmatched regression analysis (power= 0.997) (**Supplementary Table S4**). A total of 15 200 individuals aged 18 years and under were included in the ASD group based on a diagnosis of ASD and 45 600 were included in the control group, with a comparable distribution of sex, age, and insured premiums between both groups. Regarding gender distribution, 81.32% of males in both groups align with the overall gender proportion, while the percentage of females is consistent at 18.68%. The age at diagnosis exhibits a mean of 6.3 years, indicating ASD detection with different symptoms spectrum and severity which lead to a diagnosis on a broad age range (4.27 years standard deviation). Comorbidities such as ADHD, intellectual disability (IQ disability), conduct disorder, oppositional defiant disorder, and other developmental disorders show significant associations with ASD. For instance, 5.85% of individuals with ASD have ADHD, whereas only 0.01% of those without ASD exhibit this comorbidity. The presence of these comorbidities underscores the complex clinical profile of ASD cases. In terms of insured premium, the majority of individuals (99.65%) fall below NT$18,000, emphasizing a uniform socio-economic background. Individuals with ASD are more prevalent in urban areas (40.92%), with a noteworthy concentration in the highest urbanization level. Additionally, a higher proportion of ASD cases is found in hospital centers (47.75%) compared to regional and local hospitals. The study also reveals seasonal and regional variations. However, these differences are minimal, suggesting a relatively homogeneous distribution across seasons and regions in both groups.

**Table 1:** Characteristics of individuals studied at the baseline.

ASD	With		Without		*P*
Variables	n	%	n	%	
Total	15,200	25.00	45,600	75.00	
Gender					0.999
Male	12,360	81.32	37,080	81.32	
Female	2,840	18.68	8,520	18.68	
Age (years)	6.30 ± 4.27		6.37 ± 4.80		0.110
Insured premium (NT$)					<0.001
<18,000	15,176	99.84	45,410	99.58	
18,000-34,999	12	0.08	164	0.36	
≧35,000	12	0.08	26	0.06	
Attention-Deficit Hyperactivity Disorder					<0.001
Without	14,311	94.15	45,594	99.99	
With	889	5.85	6	0.01	
Intellectual disability					<0.001
Without	14,073	92.59	45,535	99.86	
With	1,127	7.41	65	0.14	
Conduct Disorder/Oppositional Defiant Disorder					<0.001
Without	15,132	99.55	45,594	99.99	
With	68	0.45	6	0.01	
Other developmental disorders					<0.001
Without	12,809	84.27	45,596	99.99	
With	2,391	15.73	4	0.01	
Childhood emotional disorder					<0.001
Without	15,118	99.46	45,600	100.00	
With	82	0.54	0	0.00	
Tourette syndrome/Tics disorders					<0.001
Without	15,085	99.24	45,592	99.98	
With	115	0.76	8	0.02	
Enuresis and encopresis					<0.001
Without	15,188	99.92	45,600	100.00	
With	12	0.08	0	0.00	
Injury					<0.001
Without	14,776	97.21	38,190	83.75	
With	424	2.79	7,410	16.25	
ISS					<0.001
<16	13,915	91.55	43,899	96.27	
≧16	1,285	8.45	1,701	3.73	
Charlson Comorbidity Index					<0.001
0	14,798	97.36	42,271	92.70	
1	346	2.28	2,885	6.33	
2	8	0.05	140	0.31	
3	35	0.23	243	0.53	
≧4	13	0.09	61	0.13	
Location					<0.001
Northern Taiwan	8,621	56.72	18,373	40.29	
Middle Taiwan	2,113	13.90	13,578	29.78	
Southern Taiwan	3,781	24.88	10,488	23.00	
Eastern Taiwan	659	4.34	2,652	5.82	
Outlets islands	26	0.17	509	1.12	
Urbanization level					<0.001
1 (The highest)	8,180	53.82	16,698	36.62	
2	5,692	37.45	18,923	41.50	
3	298	1.96	3,008	6.60	
4 (The lowest)	1,030	6.78	6,971	15.29	
Level of care					<0.001
Medical center	7,258	47.75	15,307	33.57	
Regional hospital	6,496	42.74	18,075	39.64	
Local hospital	1,446	9.51	12,218	26.79	

ASD, Autism-spectrum disorder; NT$, New Taiwan Dollars; ISS, Injury severity scale; P, Chi-square/Fisher exact test on category variables and t-test on continue variables.

### 3.2 Association between ASD and acute stress disorder and PTSD

**Figure 3:**
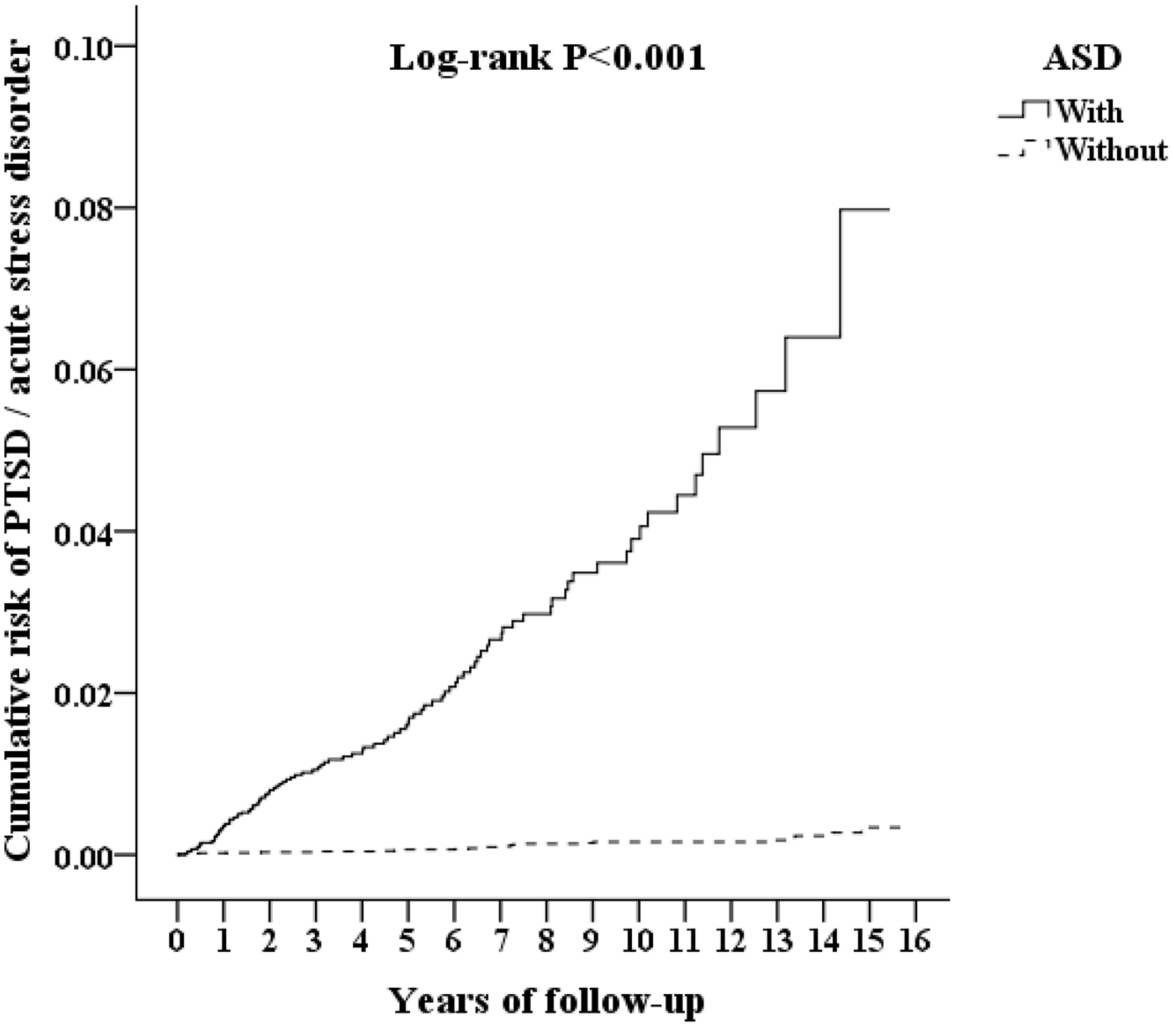
Kaplan-Meier for cumulative risk of PTSD and acute stress disorder aged 18 and less stratified by ASD with log-rank test.

In the population studied, 132 participants developed either acute stress disorder or PTSD during period of a 15-year timeframe of study: 105 (0.691% or 64.90 per 100 000 person-years) in the ASD group and 27 (0.059% or 5.38 per 100 000 person-years) in the control group. The adjusted hazard ratio for ASD was 25.611 for the studied participants (95% CI =15.913-41.232; *P* <.001) compared with the control group (**Table 2**). The Kaplan–Meier analysis revealed that the 15-year cumulative incidence of PTSD and acute stress disorder was significantly higher (log-rank test, *P* <.001, **Figure 3**) between the study participants and the controls. In the sensitivity analysis, these significant associations persisted after the exclusion of the diagnosis of acute stress disorder and/or PTSD in the first year and in the first five years (**Supplementary Table S2**).

**Table 2:** Subgroup analysis for risk of PTSD/acute stress disorder in individuals with or without ASD by using Cox regression model.

Variables	Crude HR	95% CI	95% CI	*P*	Adjusted HR	95% CI	95% CI	*P*
With ASD vs without ASD (reference)	27.647	17.843	42.837	<0.001	25.611	15.913	41.232	<0.001
Age (years)	0.886	0.857	0.916	<0.001	0.885	0.851	0.921	<0.001
ISS≧16 (reference: ISS <16)	4.204	2.943	6.004	0.001	1.286	0.868	1.904	0.212
Intellectual disability (reference: without)	2.195	1.073	4.487	0.031	1.590	0.277	1.250	0.167
Conduct Disorder/Oppositional Defiant Disorder (reference: without)	8.936	2.210	36.126	0.002	3.367	0.796	14.264	0.099
Other developmental disorders (reference: without)								
Without	Reference				Reference			
With	6.722	2.129	21.221	0.001	1.053	0.330	3.363	0.932
Childhood emotional disorder								
Without	Reference				Reference			
With	0.000	–	–	0.886	0.000	–	–	0.993
Tourette syndrome/Tics disorders								
Without	Reference				Reference			
With	0.000	–	–	0.746	0.000	–	–	0.979
Enuresis and encopresis								
Without	Reference				Reference			
With	0.000	–	–	0.950	0.000	–	–	0.997
Injury								
Without	Reference				Reference			
With	0.528	0.308	0.904	0.020	0.971	0.559	1.687	0.916
Charlson Comorbidity Index								
0	Reference				Reference			
1	0.497	0.184	1.346	0.169	0.574	0.211	1.560	0.276
2	0.000	–	–	0.955	0.000	–	–	0.971
3	2.812	0.895	8.840	0.077	2.638	0.803	8.669	0.110
≧4	1.686	0.235	12.076	0.603	1.608	0.219	11.831	0.642
Location								
Northern Taiwan	Reference				Multicollinearity with urbanization level			
Middle Taiwan	0.343	0.205	0.575	<0.001	Multicollinearity with urbanization level			
Southern Taiwan	0.820	0.550	1.224	0.332	Multicollinearity with urbanization level			
Eastern Taiwan	0.465	0.202	1.070	0.072	Multicollinearity with urbanization level			
Outlets islands	0.000	–	–	0.944	Multicollinearity with urbanization level			
Urbanization level								
1 (The highest)	2.774	1.504	5.119	0.001	1.549	0.785	3.056	0.208
2	1.353	0.717	2.553	0.351	0.937	0.481	1.824	0.847
3	0.563	0.181	1.745	0.319	0.626	0.201	1.952	0.418
4 (The lowest)	Reference				Reference			
Level of care								
Medical center	3.618	1.851	7.073	<0.001	1.622	0.809	3.249	0.174
Regional hospital	2.863	1.469	5.579	0.002	1.257	0.596	2.652	0.550
Local hospital	Reference				Reference			

PTSD, Post-traumatic stress disorder; HR, Hazard ratio; CI, Confidence interval, Adjusted HR, Adjusted variables listed in the **Table 1**.

Furthermore, given that past literature has indicated ADHD as one of the factors increasing the risk of PTSD (43), in addition to age and sex, we included ADHD comorbidity in the matching criteria in another regression analysis. (**Supplementary Table S4**) The results revealed that ASD cases developing PTSD had an adjusted HR of 22.184, with a 95% CI of 12.106~37.195, P < 0.001. This underscores that even after matching for ADHD comorbidity, the significant correlation with a higher risk persists.

### 3.3 Association between different frequencies of psychiatric visits and acute stress disorder and PTSD

The association between ASD and the risk of PTSD or acute stress disorder was analyzed in three subgroups: 1. Acute stress disorder or PTSD, 2. PTSD only, and 3. Acute stress disorder only (**Table 3**). The adjusted hazard ratio for the <3 visits group is 28.906, for the 3-5 visits group is 15.202, and for the >=6 visits group is 14.532. In each subgroup, ASD was associated with a higher risk of PTSD and/or acute stress disorder. Individuals with ASD who visit more frequently may experience a slight reduction in this risk.

**Table 3:** Factors of PTSD/acute stress disorder among different frequency of psychiatry visits by using Cox regression.

Events subgroup	Frequency of psychiatry visits	Events	Rate (per 10^5^ PYs)	Adjusted HR	95% CI	95% CI	*P*
PTSD/acute stress disorder	Without ASD	27	5.38	Reference			
	With ASD	105	64.90	25.611	15.913	41.232	<0.001
	With ASD, < 3 visits per year	51	96.14	28.906	23.592	61.160	<0.001
	With ASD, 3-5 visits per year	31	50.63	15.202	12.409	32.144	<0.001
	With ASD, ≧6 visits per year	23	48.41	14.532	11.863	30.727	<0.001
PTSD	Without ASD	7	1.39	Reference			
	With ASD	19	11.74	23.403	8.553	64.110	<0.001
	With ASD, < 3 visits per year	11	20.74	40.887	15.117	113.489	<0.001
	With ASD, 3-5 visits per year	6	9.80	19.286	7.136	53.462	<0.001
	With ASD, ≧6 visits per year	2	4.21	8.271	3.062	22.906	<0.001
Acute stress disorder	Without ASD	20	3.98	Reference			
	With ASD	86	53.16	27.360	15.847	47.255	<0.001
	With ASD, < 3 visits per year	40	75.40	32.837	22.496	67.117	<0.001
	With ASD, 3-5 visits per year	25	40.83	17.756	12.167	36.270	<0.001
	With ASD, ≧6 visits per year	21	44.20	19.223	13.172	39.268	<0.001

PTSD, Post-traumatic stress disorder; PYs, Person-years; Adjusted HR, Adjusted Hazard ratio; Adjusted for the variables listed in **Table 1**.; CI, Confidence interval.

## 4 Discussion

This study presents several noteworthy findings. First, we established that youths with ASD have a more than 20-fold increased risk of developing acute stress disorder and PTSD. Second, to resolve the influences of protopathic bias, we conducted the sensitivity analysis revealed the association between ASD and acute stress disorder and PTSD even after exclusion of diagnoses within the first year and in the first five years. Third, ASD is associated with acute stress disorder and PTSD for PTSD-alone, acute stress disorder-alone, or combination of PTSD and acute stress disorder. Fourth, increased frequency of psychiatric visits is associated with a less higher risk of acute stress disorder and PTSD. For this finding, we hypothesize that a higher frequency of psychiatric visits might be an indicator of more regular and intensive treatment and care for ASD patients, and these might be associated with better resilience to traumatic events the patients that experienced. Our nationwide cohort study differs from former studies in that we used a wide-ranging national health insurance registry to identify the participants with a formal diagnosis of ASD. Further, our study was across the period of a 15-year timeframe to evaluate the occurrence of acute stress disorder and PTSD. Therefore, this study provides stronger evidence of the relationship between ASD and acute stress disorder and PTSD. To the best of our knowledge, this is the first study investigating the association between ASD and the risk of acute stress disorder and PTSD in a population-based cohort within a 15-year timeframe.

The retrospective design of the study was chosen due to practical considerations and the nature of the data available in the NHIRD in Taiwan. Retrospective studies are often employed when longitudinal data is already accessible, and obtaining prospective data is challenging or resource intensive. In this case, utilizing existing data allowed for a large sample size and an extended follow-up period, providing valuable insights into the association between ASD and the risk of developing PTSD and acute stress disorder. The choice of study design was driven by the feasibility and accessibility of the data, ensuring a comprehensive examination of the research question within the available constraints.

We based our decision to match on age and gender on considerations outlined by Brazauskas and Logan (44), emphasizing the importance of a matched study design in cases requiring reduced individuals or exhibiting a large degree of heterogeneity (44). This choice aimed to optimize the validity and reliability of our study results.

The ASD cohort in our study, in contrast to earlier studies (1, 3), shows lower comorbidity rates. For instance, previous literature indicated higher comorbidity rates for ASD, such as ADHD ranging from 28% to 44%, while our study reports a rate of 5.85%. The comorbidity rate with intellectual disability was 21-45% in previous literature, but our study found a rate of 7.41%. Similarly, the comorbidity rate with learning disabilities was 23.5% in previous literature and 15.73% in this study, showing closer alignment. These lower comorbidity rates might relate to the timeframe of the study, conducted between 2000 and 2015 when the diagnostic system in Taiwan used DSM-IV-TR. During this period, ASD and ADHD diagnoses were mutually exclusive, leading clinicians to preferentially register a more severe ASD diagnosis in NHIRD to enhance access to social welfare or special education resources. This practice may have resulted in an underestimation of ADHD comorbidity rates. Concerning intellectual disability, the DSM-IV-TR required standardized intelligence testing with results falling below two standard deviations for diagnosis. However, due to the severity of ASD or the age of the patients, standardized intelligence testing might not have been feasible, leading to potential underestimation of intellectual disability comorbidity rates. Therefore, the lower comorbidity rates in our study are likely related to the diagnostic system used during the study period.

The diagnostic criteria of PTSD in DSM defined trauma as “exposure to actual or threatened death, serious injury, or sexual violence (9, 10).” However, several studies have attempted to broaden the experience of psychological trauma (non-DSM traumatic events), such as loss, work, relationships, academic achievements, environment, life transitions, or physical struggles, through a questionnaire-based study on participants with ASD (5, 19, 29). Individuals with ASD may develop PTSD symptoms after encountering such non-DSM traumatic events, implying that stressful events in everyday life may result in the development of PTSD in ASD adults (18).

The mechanism between ASD and PTSD as well as the associated risk factors are still under investigation. A hypothetical model assumed that ASD may influence the experience of trauma at different levels, moderating the event encountered, experiences appraised harmful, and the risk of developing PTSD or other negative outcomes such as affective disorders (45).

Children and adolescents with ASD are prone to experiencing bullying or peer victimization (46). An article of systematic review revealed prevalence rates of 33% for physical bullying, 50% for verbal bullying, and 31% for relational bullying in ASD children (47). These encounters may be linked to subsequent manifestations of school refusal (48). Another study identified correlations between bullying victimization among ASD children and ASD symptoms such as social and communication deficits, internalizing behaviors, and condition of participation in integrated inclusive school settings (49). Even in cases of high-functioning ASD children or with Asperger’s syndrome, bullying remains prevalent (50), with over half of such cases reporting instances of victimization in a study (51).

Higher suicidal thoughts or behaviors were observed in children with ASD (6). A review article summarized that ASD individuals, with unique characteristic of sensation, perception, social awareness and cognition, may experience psychosocial events as traumatic compared with general population, such as unusual fears, difficulties with sensory overstimulation, changes in routine, or social demands (52). Various of studies investigated on the possible shared symptomology and mechanism between ASD and PTSD, including memory problems (18), brooding rumination (53), cognitive rigidity (54, 55), emotional dysregulation (56, 57), irritability and aggression (58, 59), and avoidance (60, 61). Furthermore, other studies have indicated that one mediator contributing to increased suicide rates in the PTSD population is the experience of guilt associated with trauma (62, 63). Additionally, individuals with ASD are more prone to experiencing self-conscious emotions such as guilt and shame compared to the typically-developed population (64). It remains to be further explored whether the experience of guilt could potentially serve as a mediating factor in the higher occurrence of PTSD within the ASD youths.

On the aspect of gender difference, a study revealed that female ASD patients are at higher risk of exposure to non-DSM traumatic events and development of PTSD symptoms than males (22). However, another study showed no geneder difference in developing PTSD symptoms in ASD individuals who exposed to non-DSM traumatic events (18). In our study, no significant difference was observed in the occurrence of acute stress disorder or PTSD between males and females. Compared to males, female ASD individuals may be diagnosed in older age, and have tendency of camouflaging their autistic features and using compensatory behaviors (65). The gender may be a potential factor to affect the developing acute stress disorder and PTSD in ASD individuals, but its role is still unclear. The discrepancy on gender difference of previous studies and our work may indicate that a certain group of ASD individual, possibly with trauma experience and PTSD symptoms, has been beyond the scope of PTSD diagnostic criteria of DSM. The undiagnosed group of ASD patients with PTSD symptoms may be potentially neglected (66).

Lastly, the Spencer et al. (43) meta-analysis suggests an elevated risk of individuals with ADHD developing PTSD. Our study, constrained by diagnostic system limitations, reports a relatively low comorbidity rate of ADHD in individuals with ASD, hindering a full representation of this population. However, when including ADHD in matching criteria, the heightened risk persists, affirming that ASD independently associates with an increased development of PTSD/acute stress disorder. Nevertheless, Spencer et al. (43) highlights that the heightened PTSD risk in individuals with ADHD goes beyond trauma exposure, hinting at a potential neurobiological basis. In contrast, our discussion above of ASD and PTSD links ASD symptomatology to an increased likelihood of trauma exposure, potentially elevating PTSD risk. These differing pathways suggest that ASD and ADHD may influence PTSD risk differently, exceeding our study’s scope. Additionally, past studies on ADHD and PTSD often overlooked ASD comorbidity, emphasizing the need for further research to unravel the intricate relationship among ASD, ADHD, and PTSD.

### 4.1 Limitations

This study has several limitations. First, The incidence of ASD may be underestimated as our study enrolled only medical help-seeking patients. Parents or teachers might observe developmental delays or signs of ASD in children, but not all are brought to hospitals or clinics due to potential social stigma associated with psychiatric evaluation.

Second, the diverse age of ASD diagnosis, ranging from early childhood to adolescence, is influenced by the spectrum nature and severity of symptoms. More severe cases are diagnosed early, while milder cases may receive a diagnosis later. Our large sample reflects this spectrum. Employing age and gender matching, considering higher male prevalence, enhances result power but presents limitations like sample size loss and selection bias. Propensity score matching and sensitivity analysis address these challenges, emphasizing our commitment to accurate and valid results.

Third, in our cohort of youths with ASD, comorbidity rates were notably lower than expected based on previous studies (1, 3). This discrepancy is likely attributed to the use of the DSM-IV-TR diagnostic system during the study period, as discussed in the previous section, resulting in lower rates of comorbidities in our study. Additionally, our study observed a lower comorbidity rate of ADHD in the sample, despite previous literature indicating a higher risk of developing PTSD in individuals with ADHD. By incorporating ADHD comorbidity in our another analysis, we identified a heightened correlation, suggesting that ASD itself is associated with an increased risk of PTSD/acute stress disorder. However, the applicability of these comorbidity rates from our sample to the entire population has certain limitations.

Fourth, the increased number of visits by ASD cases may introduce detection bias and heightened diagnoses and comorbidities. Detection bias persists in this study. In **Table 3**, individuals with more frequent visits may experience a slightly lower risk. Thus, we interpret that the risk of developing PTSD/acute stress disorder increases regardless of visit frequency, with a slightly lower increase for those with more visits, possibly linked to ASD patients receiving comprehensive medical care during frequent visits, mitigating this risk.

Fifth, this study did not investigate and analyze other factors that could contribute to the development of PTSD, such as domestic violence, peer bullying at school, single-parent households, etc. This limitation is primarily associated with the constraints of the health insurance database’s covered content. Additionally, excessive matching variables may lead to overmatching, potentially obscuring existing risks (67). Sixth, the health insurance database lacks information on whether standardized diagnostic tools, like the Autism Diagnostic Observation Schedule (ADOS) or the Autism Diagnostic Interview-Revised (ADI-R), were used for ASD diagnosis, or data on the severity of ASD and associated psychological assessment results, which could impact the risk of developing PTSD or acute stress disorder.

Finally, our study did not differentiate trauma types or assess the severity of ASD and PTSD adequately within the NHIRD. This lack of specificity may lead to varying risks across different trauma-exposed groups. Additionally, our reliance on DSM-IV-TR criteria for PTSD diagnosis by psychiatrists excluded cases where individuals with ASD experienced non-DSM-defined traumas, potentially displaying PTSD symptoms without meeting diagnostic criteria. We also overlooked individuals with concerns aligning with complex PTSD in ICD-11, emphasizing chronic, repetitive, and prolonged traumas affecting emotional regulation, self-identity, and relational capacities (68). These omissions suggest the need for future research to encompass these considerations.

## 5 Conclusions

Through a nationwide population-based cohort study in Taiwan, we assessed the risk of acute stress disorder and PTSD among children and adolescents with ASD. We established that children and adults with ASD have a more than 20-fold increased risk of developing acute stress disorder and PTSD. We sincerely hope this study can be used as a reference to examine patients with ASD and design a therapeutic plan from the perspective of trauma exposure and post-trauma process in daily practice.

## Data availability statement

The data analyzed in this study is subject to the following licenses/restrictions: Data are available from the National Health Insurance Research Database (NHIRD) published by Taiwan National Health Insurance (NHI) Bureau. Due to legal restrictions imposed by the government of Taiwan in relation to the “Personal Information Protection Act”, data cannot be made publicly available. Requests for data can be sent as a formal proposal to the NHIRD. Requests to access these datasets should be directed to http://nhird.nhri.org.tw.

## Ethics statement

The studies involving humans were approved by the institutional review board of the Tri-Service General Hospital (IRB No.2-107-05-026). The studies were conducted in accordance with the local legislation and institutional requirements. The ethics committee/institutional review board waived the requirement of written informed consent for participation from the participants or the participants’ legal guardians/next of kin because identities of individuals in the NHIRD were completely encrypted to protect privacy.

## Author contributions

SL: Visualization, Writing – original draft, Writing – review & editing. WC: Data curation, Investigation, Methodology, Writing – original draft. CC: Formal analysis, Resources, Software, Validation, Visualization, Writing – original draft. NT: Conceptualization, Funding acquisition, Methodology, Project administration, Supervision, Writing – original draft.

## Supplementary material

Supplementary Figure 1 The estimated statistical power of the ratio for the case to control groups (Figure S1a adapted from Woodward 2013*). Figure S1b showed our statistical analysis showed the power was approximate to 1.0 while match ratio was 1:3 . * Woodward M (2013). Epidemiology: Study Design and Data Analysis, 3rd Edition. United Kingdom, Chapman and Hall/CRC.
