# Supplementary material for: Increased risk of acute stress disorder and post-traumatic stress disorder in children and adolescents with autism spectrum disorder: a nation-wide cohort study in Taiwan
Source: Front Psychiatry. 2024 Jan 31;15:1329836. doi: 10.3389/fpsyt.2024.1329836 (PMC10864464; doi:10.3389/fpsyt.2024.1329836)
Supplement: Supplementary Figure 1 — The estimated statistical power of the ratio for the case to control groups (Figure S1a adapted from Woodward 2013*). Figure S1b showed our statistical analysis showed the power was approximate to 1.0 while match ratio was 1:3 . * Woodward M (2013). Epidemiology: Study Design and Data Analysis, 3rd Edition. United Kingdom, Chapman and Hall/CRC. [file Image_1.pdf]

Figure S1a.

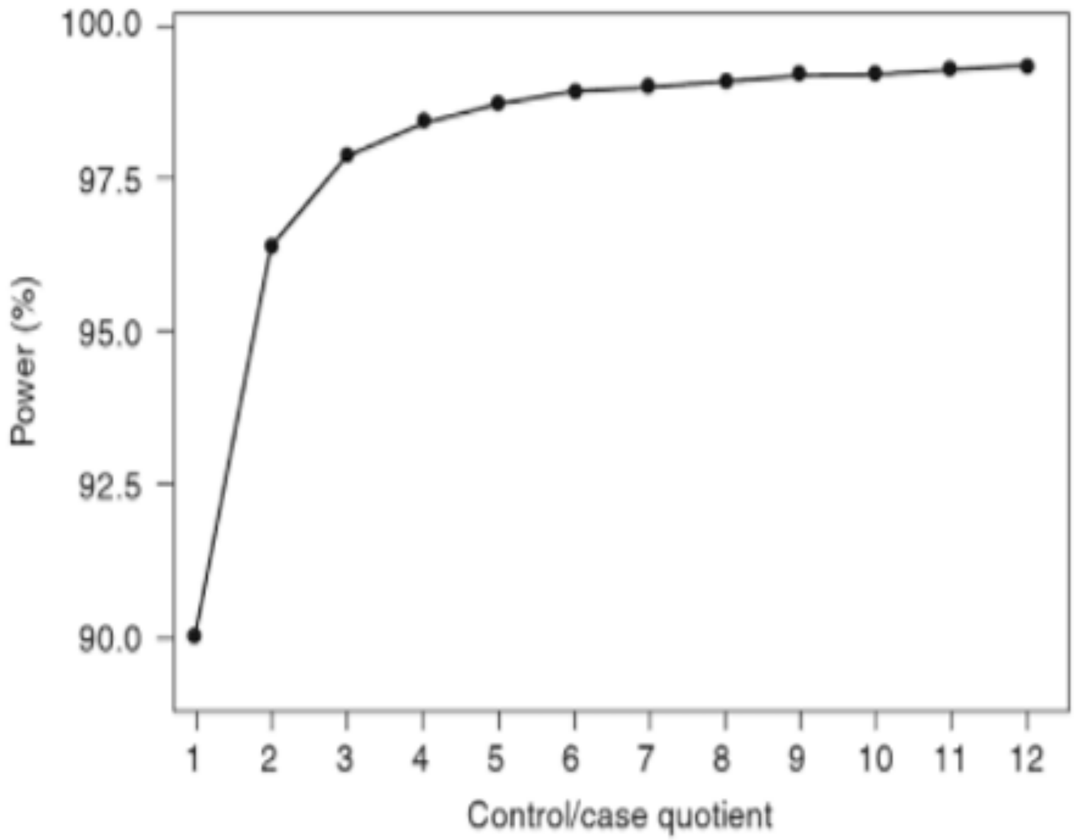

Figure S1b.

```
. sampsi 0.69 0.06, alpha(0.05) n1(15200) ratio(3)

Estimated power for two-sample comparison of proportions

Test Ho: p1 = p2, where p1 is the proportion in population 1
                    and p2 is the proportion in population 2
Assumptions:

         alpha =   0.0500  (two-sided)
            p1 =   0.6900
            p2 =   0.0600
sample size n1 =    15200
            n2 =    45600
         n2/n1 =     3.00

Estimated power:

         power =   1.0000
```
