# Supplementary material for: Increased risk of acute stress disorder and post-traumatic stress disorder in children and adolescents with autism spectrum disorder: a nation-wide cohort study in Taiwan
Source: Front Psychiatry. 2024 Jan 31;15:1329836. doi: 10.3389/fpsyt.2024.1329836 (PMC10864464; doi:10.3389/fpsyt.2024.1329836)
Supplement: Supplementary file 2 [file Table_1.docx]

Supplementary Material

# Supplementary Tables

Supplementary Table S1. ICD-9-CM codes in the diagnoses and clinical situations in this study.

| Autism spectrum disorder | 299 |
| --- | --- |
| Posttraumatic stress disorder | 309.81 |
| Acute stress disorder | 308 |
| Attention-deficit hyperactivity disorder | 314 |
| Intelligence disability | 317-319, V62.89 |
| Conduct disorder / Oppositional defiant disorder | 312 |
| Other developmental disorders | 315 |
| Childhood emotional disorder | 313 |
| Tourette syndrome / Tics disorders | 307.2 |
| Enuresis and encopresis | 307.6-307.7 |
| Injury | 800-999, E800-E999 |

ICD-9-CM: International Classification of Diseases, 9th Revision, Clinical Modification.

Supplementary Table S2. Sensitivity test for factors of PTSD / acute stress disorder by using Cox regression.

| Sensitivity test |  | With | | | Without *(Reference)* | | | With *vs.* without *(Reference)* | | | |
| --- | --- | --- | --- | --- | --- | --- | --- | --- | --- | --- | --- |
|  | Events subgroup | Events | PYs | Rate (per 10^5^ PYs) | Events | PYs | Rate (per 10^5^ PYs) | Adjusted HR | 95% CI | 95% CI | *P* |
| Overall | PTSD / acute stress disorder | 105 | 161,785.99 | 64.90 | 27 | 502,242.85 | 5.38 | 25.611 | 15.913 | 41.232 | <0.001 |
|  | PTSD | 19 | 161,785.99 | 11.74 | 7 | 502,242.85 | 1.39 | 23.403 | 8.553 | 64.110 | <0.001 |
|  | Acute stress disorder | 86 | 161,785.99 | 53.16 | 20 | 502,242.85 | 3.98 | 27.360 | 15.847 | 47.255 | <0.001 |
| In the first year excluded | PTSD / acute stress disorder | 84 | 155,370.45 | 54.06 | 22 | 497,443.51 | 4.42 | 31.671 | 18.669 | 53.748 | <0.001 |
|  | PTSD | 16 | 155,370.45 | 10.30 | 7 | 497,443.51 | 1.41 | 20.643 | 7.317 | 58.310 | <0.001 |
|  | Acute stress disorder | 68 | 155,370.45 | 43.77 | 15 | 497,443.51 | 3.02 | 38.060 | 20.347 | 71.234 | <0.001 |
| In the first 5 years excluded | PTSD / acute stress disorder | 40 | 106,244.06 | 37.65 | 15 | 426,915.72 | 3.51 | 33.731 | 16.850 | 67.570 | <0.001 |
|  | PTSD | 7 | 106,244.06 | 6.59 | 4 | 426,915.72 | 0.94 | 44.449 | 9.513 | 208.534 | <0.001 |
|  | Acute stress disorder | 33 | 106,244.06 | 31.06 | 11 | 426,915.72 | 2.58 | 35.247 | 15.987 | 77.785 | <0.001 |

PYs = Person-years; Adjusted HR = Adjusted Hazard ratio: Adjusted for the variables listed in Table 1.; CI = Confidence interval.

| **Supplementary Table S3. Characteristics of study in the baseline, unmatched** | | | | | | | |
| --- | --- | --- | --- | --- | --- | --- | --- |
| **ASD** | **Total** |  | **With** |  | **Without, unmatched** | | ***P*** |
| **Variables** | **n** | **%** | **n** | **%** | **n** | **%** |  |
| **Total** | 327,275 |  | 15,200 | 4.64 | 312,075 | 95.36 |  |
| **Gender** |  |  |  |  |  |  | < 0.001 |
| Male | 168,661 | 51.53 | 12,360 | 81.32 | 156,301 | 50.08 |  |
| Female | 158,614 | 48.47 | 2,840 | 18.68 | 155,774 | 49.92 |  |
| **Age (years)** | 2.46 ± 5.81 |  | 6.30 ± 4.27 |  | 2.27 ± 5.81 |  | < 0.001 |
| **Insured premium (NT$)** |  |  |  |  |  |  | < 0.001 |
| < 18,000 | 206,849 | 63.20 | 15,176 | 99.84 | 191,673 | 61.42 |  |
| 18,000 - 34,999 | 75,703 | 23.13 | 12 | 0.08 | 75,691 | 24.25 |  |
| ≧ 35,000 | 44,723 | 13.67 | 12 | 0.08 | 44,711 | 14.33 |  |
| **ISS** |  |  |  |  |  |  | < 0.001 |
| < 16 | 309,471 | 94.56 | 13,915 | 91.55 | 295,556 | 94.71 |  |
| ≧ 16 | 17,804 | 5.44 | 1,285 | 8.45 | 16,519 | 5.29 |  |
| **ADHD** |  |  |  |  |  |  | < 0.001 |
| Without | 323,221 | 98.76 | 14,311 | 94.15 | 308,910 | 98.99 |  |
| With | 4,054 | 1.24 | 889 | 5.85 | 3,165 | 1.01 |  |
| **IQ disability** |  |  |  |  |  |  | < 0.001 |
| Without | 324,357 | 99.11 | 14,073 | 92.59 | 310,284 | 99.43 |  |
| With | 2,918 | 0.89 | 1,127 | 7.41 | 1,791 | 0.57 |  |
| **CD / ODD** |  |  |  |  |  |  | < 0.001 |
| Without | 325,143 | 99.35 | 15,132 | 99.55 | 310,011 | 99.34 |  |
| With | 2,132 | 0.65 | 68 | 0.45 | 2,064 | 0.66 |  |
| **Other developmental disorders** |  |  |  |  |  |  | < 0.001 |
| Without | 324,661 | 99.20 | 12,809 | 84.27 | 311,852 | 99.93 |  |
| With | 2,614 | 0.80 | 2,391 | 15.73 | 223 | 0.07 |  |
| **Childhood emotional disorder** |  |  |  |  |  |  | < 0.001 |
| Without | 327,033 | 99.93 | 15,118 | 99.46 | 311,915 | 99.95 |  |
| With | 242 | 0.07 | 82 | 0.54 | 160 | 0.05 |  |
| **Tourette syndrome / Tics disorders** |  |  |  |  |  |  | < 0.001 |
| Without | 327,073 | 99.94 | 15,085 | 99.24 | 311,988 | 99.97 |  |
| With | 202 | 0.06 | 115 | 0.76 | 87 | 0.03 |  |
| **Enuresis and encopresis** |  |  |  |  |  |  | < 0.001 |
| Without | 327,239 | 99.99 | 15,188 | 99.92 | 312,051 | 99.99 |  |
| With | 36 | 0.01 | 12 | 0.08 | 24 | 0.01 |  |
| **Injury** |  |  |  |  |  |  | < 0.001 |
| Without | 296,710 | 90.66 | 14,776 | 97.21 | 281,934 | 90.34 |  |
| With | 30,565 | 9.34 | 424 | 2.79 | 30,141 | 9.66 |  |
| **CCI** |  |  |  |  |  |  | < 0.001 |
| 0 | 312,448 | 95.47 | 14,798 | 97.36 | 297,650 | 95.38 |  |
| 1 | 8,873 | 2.71 | 346 | 2.28 | 8,527 | 2.73 |  |
| 2 | 4,320 | 1.32 | 8 | 0.05 | 4,312 | 1.38 |  |
| 3 | 1,236 | 0.38 | 35 | 0.23 | 1,201 | 0.38 |  |
| ≧4 | 398 | 0.12 | 13 | 0.09 | 385 | 0.12 |  |
| **Season** |  |  |  |  |  |  | < 0.001 |
| Spring | 74,272 | 22.69 | 3,769 | 24.80 | 70,503 | 22.59 |  |
| Summer | 95,597 | 29.21 | 4,030 | 26.51 | 91,567 | 29.34 |  |
| Autumn | 86,591 | 26.46 | 4,136 | 27.21 | 82,455 | 26.42 |  |
| Winter | 70,815 | 21.64 | 3,265 | 21.48 | 67,550 | 21.65 |  |
| **Location** |  |  |  |  |  |  | < 0.001 |
| Northern Taiwan | 98,483 | 30.09 | 8,621 | 56.72 | 89,862 | 28.80 |  |
| Middle Taiwan | 85,677 | 26.18 | 2,113 | 13.90 | 83,564 | 26.78 |  |
| Southern Taiwan | 89,561 | 27.37 | 3,781 | 24.88 | 85,780 | 27.49 |  |
| Eastern Taiwan | 36,450 | 11.14 | 659 | 4.34 | 35,791 | 11.47 |  |
| Outlets islands | 17,104 | 5.23 | 26 | 0.17 | 17,078 | 5.47 |  |
| **Urbanization level** |  |  |  |  |  |  | < 0.001 |
| 1 (The highest) | 90,255 | 27.58 | 8,180 | 53.82 | 82,075 | 26.30 |  |
| 2 | 89,798 | 27.44 | 5,692 | 37.45 | 84,106 | 26.95 |  |
| 3 | 69,999 | 21.39 | 298 | 1.96 | 69,701 | 22.33 |  |
| 4 (The lowest) | 77,223 | 23.60 | 1,030 | 6.78 | 76,193 | 24.41 |  |
| **Level of care** |  |  |  |  |  |  | < 0.001 |
| Hospital center | 108,710 | 33.22 | 7,258 | 47.75 | 101,452 | 32.51 |  |
| Regional hospital | 110,388 | 33.73 | 6,496 | 42.74 | 103,892 | 33.29 |  |
| Local hospital | 108,177 | 33.05 | 1,446 | 9.51 | 106,731 | 34.20 |  |
| ***P:* Chi-square / Fisher exact test on category variables and t-test on continue variables** | | | |  |  |  |  |

|  | Supplementary Table S4. Factors of PTSD / acute stress disorder by using Cox regression | | | | | | | | | |
| --- | --- | --- | --- | --- | --- | --- | --- | --- | --- | --- |
| **With ASD *vs.***  **Without ASD** *(Reference)* | | **Crude HR** | **95% CI** | **95% CI** | ***P*** | **aHR** | **95% CI** | **95% CI** | ***P*** | **Post hoc Power** |
| 3-fold propensity score matching by gender, age, ADHD and index date | | 24.235 | 15.576 | 39.801 | < 0.001 | 22.184 | 12.106 | 37.195 | < 0.001 | 0.999 |
| 3-fold propensity score matching by gender, age, and index date | | 27.647 | 17.843 | 42.837 | < 0.001 | 25.482 | 15.850 | 40.969 | < 0.001 | 0.999 |
| Unmatched | | 75.104 | 32.319 | 110.561 | < 0.001 | 35.654 | 9.142 | 97.180 | < 0.001 | 0.997 |
|  | **CI = confidence interval; aHR = Adjusted HR: Adjusted variables listed in Table 3.** | | | | | | | | | |
